# Desalination of Neutral Amino Acid Solutions in an Electromembrane System

**DOI:** 10.3390/membranes12070665

**Published:** 2022-06-28

**Authors:** Tatiana Eliseeva, Anastasiia Kharina

**Affiliations:** Department of Analytical Chemistry, Voronezh State University, 1 Universitetskaya pl., 394018 Voronezh, Russia; aukharina@gmail.com

**Keywords:** amino acid, transport, ion-exchange membrane, electrodialysis, electrodeionization, desalination, limiting current density, current–voltage curve

## Abstract

This article’s main focus is to highlight significant aspects of amino acid solution demineralization. The main part of the amino acid production method requires the provision of downstream treatment solutions for the process of desalination. Electrodialysis (ED) and electrodeionization (EDI) are prospective technologies for such treatment. The article presents a brief review of the first studies and current research on electromembrane desalination of amino acid solutions as well as the analysis of some electrochemical features for the mineral salt–amino acid system (model solution) in an ED process based on the experimental results. The influence of various factors on the desalination of neutral amino acid-containing solutions and on target product losses in this process is estimated. The behavior of aliphatic (alanine) and aromatic (phenylalanine) amino acids in the electromembrane system is considered in mixed solutions with inorganic electrolytes. The influence of various mineral cations (Na^+^, K^+^ and NH_4_^+^) and anions (NO_3_^−^, SO_4_^2−^, Cl^−^) on the features of the transport and current–voltage characteristics of ion-exchange membranes in the electrodialysis of phenylalanine- and alanine-containing solutions is considered. A comparative analysis of the desalination parameters of AA solutions in electrodialysis with the following pairs of heterogeneous MA-41/MK-40, MA-40/MK-40 and homogeneous AMT/CMT membranes is carried out. The minimum amount of amino acid loss along with rather high values of the degree of desalination are revealed in electrodialysis with polypropylene spacers in comparison with EDI, ED with a copolymer of styrene and divinylbenzene as spacer, as well as ED with a smooth deionization channel. At the same time, EDI is the most promising method to reach the highest desalination degree in the considered range of mineral salt content.

## 1. Introduction

Electrodialysis is an efficient separation method which has been successfully used for brackish water desalination, wastewater purification, whey processing, recovery and the concentration of valuable compounds in biotechnology etc. [1,2,3,4,5,6,7,8,9,10]. Electrodialysis can be an alternative technique for nutrient recovery [11,12,13,14]. A very promising field of ED application is amino acid production in biorefinery [15,16].

Amino acids are referred to as zwitterlytes—the most important group of organic ampholytes for all living organisms [17]. They can exist as bipolar ions (zwitterions), cations and/or anions in aqueous solutions depending on the pH value. Amino acid at a pH value close to the isoelectric point (designated as pI) has no net electric charge. For the most part, it is in the form of zwitterions, which cannot migrate under the influence of an electric field; therefore, the idea to separate strong electrolytes (cations and anions) and amino acids in their zwitter-form using a gradient of potential as a driving force is rather long-standing. The electrodialysis with “charged membranes” was used for amino acid recovery from a mineralized solution at an early stage of the development of this method. Tage Astrup and Agnete Stage were the first to have proposed an electrodialysis procedure for amino acid solution desalting [18]. They considered the desalting of glycine, arginine and glutamic acid solutions in a batch mode, estimating their losses in the process. The loss of glutamic acid was 4–25% depending on the membrane used, but for neutral and basic amino acids, it reached up to 50–90%. The authors explained this as being due to the insufficient quality of membranes. They have applied cellophane and cellophane impregnated with a phenylenediamine resin membrane developed by G.A. Gilbert and A.J. Swallow [19]. The experiments with an “electropositive” impregnated cellophane membrane provided better recovery of amino acids. No data on the pH value of demineralized solutions have been indicated. Di Benedetto A.T. and Lightfoot E.N. [20] studied the dependence of glycine (neutral amino acid) and chloride-ion separation efficiency based on the pH value in ED and they found that at a pH close to the pI, the transfer of glycine through the ion-exchange membrane was minimal and reached 6.1% of the feed concentration. A deviation of the pH value into the acidic or basic range increased the transport of amino acids from the desalination compartment. Therefore, at pH 9 the losses of glycine were 15.7% and at pH 11.3 they were 51%. The other researchers have confirmed that the minimal transfer of amino acid through the anion- and cation-exchange membranes can be observed at the solution’s pH value in the deionization compartments, which is close to the pI [21]. However, the diffusion of amino acids through the membranes was indicated as a problem, decreasing the efficiency of desalination.

J.D. Blainey and H.J. Yardley [22] studied the desalination of various amino acid mixtures (glycine, alanine, phenylalanine, valine, lysine, arginine, glutamic and aspartic acid) in a three-compartment electrodialyzer with an industrial Permaplex A.1 anion-exchange membrane from the anode side and a Permaplex C.10 cation-exchange membrane (Permutit Ltd., London, UK) from the cathode side. These membranes have provided better results for amino acid recovery and desalination degree than earlier work. The authors used sulphuric acid (0.1 M) and sodium hydroxide (0.2 M) solutions in cathode and anode compartments, respectively. The results were obtained at a single initial value of current density in a batch mode. Loss of various amino acids in the study of Blainey and Yardley was explained by their acidic−basic properties and relative mobility. The demineralization process was carried out as a pretreatment stage for the chromatographic analysis.

A.M. Peers [23] also considered the electromembrane desalination of amino acids with different isoelectric points: aspartic acid, alanine, lysine and arginine. Based on the analysis of the obtained data, he has come to the conclusion that zwitterions of amino acids, along with cations and anions, are partially transferred through membranes in electrodialysis. The loss of amino acids was minimal when using a three-compartment apparatus with anion- and cation-exchange membranes located on the sides of the anode and cathode; there was a strong acid in the anode compartment and a strong base in the cathode compartment. Thus, Peers used the scheme of a three-compartment cell similar to [22] but after comparison of different solutions in the electrode compartments, he decided to fill the anode compartment with acid solution and the cathode compartment with base solution. The diffusion of acid through the anion-exchange membrane was restricted but it prevented amino acid anion migration through the anion-exchange membrane because they recharged into zwitterions and cations. Similarly, the diffusion of alkali through the membrane from the cathode compartment led to the recharging of amino acid cations into zwitterions and anions that could not migrate through the cation-exchange membrane. This idea to provide “acidic/basic barriers” helps to decrease ampholyte losses in ED extraction but its implementation requires additional chemical reagent consumption.

Reagent-free enhancement of the efficiency of the demineralization process is possible if the special dependence of amino acid transport in electrodialysis on the current density and the choice of a proper value of current density are taken into account. The main features of amino acid transport in electrodialysis are an increase in flux through the ion-exchange membrane with an increase in the current density in underlimiting mode due to the existence of cations and anions in the solution. The main features of amino acid transport in electrodialysis are an increase in flux through the ion-exchange membrane with an increase in the current density in underlimiting mode due to the existence of cations and anions in the solution even at pH=pI, the occurrence of barrier effect and the effect of facilitated (stimulated) transport in overlimiting mode [24,25,26].

Barrier effect makes it possible to improve the performance of electromembrane desalination for amino acid-containing solutions [25]. Such an effect has been found primarily in ED of mannitol (organic ampholyte) solution [27]. Then it was applied for the deep demineralization of neutral amino acid solutions [28], and the dependence of neutral amino acid flux through the membranes on the current density has been determined [29]. The essence of the barrier effect phenomenon lies in the fact that when reaching the limiting current density, water splitting occurs at the surfaces of membranes in the desalting compartments and the excess of current is transferred by hydrogen ions through the cation-exchange membrane and by hydroxyl ions through the anion-exchange membrane. Therefore, the “barriers” of hydroxyl and hydrogen ions appearing near the surface of cation- and anion-exchange membranes, respectively, in the desalting compartment can restrict the transport of an ampholyte. When cations and zwitterions make contact with hydroxyl ions near the surface of the cation-exchange membrane, they are recharged into anions and restricted in the desalting compartment. Similarly, hydrogen ions near the surface of the anion-exchange membrane in the desalting compartment become a barrier for amino acid transport through the anion-exchange membrane. The total action of two “barriers” at the interphase boundaries of membranes has been called the “circulation effect” [26]. It is necessary to make a point that the circulation effect is a special feature in the transport of zwitterlites, which can exist in the form of cations, zwitterions and anions depending on pH and change the direction of their migration in ED. However, the more common term, “barrier effect”, is suitable for all ampholytes. Barrier effect leads to a decrease in ampholyte migration in the form of cations and/or anions through the cation- and/or anion-exchange membrane, respectively, and mitigates losses of the target product in its electrodialysis demineralization [30]. The barrier effect phenomenon has been directly approved for various amino acid solutions by the method of laser interferometry [31]. It has been shown that after reaching the limiting current density, the surface concentration of amino acids at the boundary membrane solution increases because of the prompt accumulation of zwitterions at the interphase boundary caused by water splitting and the participation of hydrogen and hydroxyl ions in current transfer through the cation- and anion-exchange membranes, respectively.

It bears mentioning that the dependence of amino acid flux through the membrane on the current density presented in [29] is incomplete. The discussed range of current density is rather short. If we were to apply higher values of current density (two times more than the limiting one) in electrodialysis than those reported by the authors, one can observe the following increase in amino acid flux [25,26]. This effect of facilitated transport discussed in [25,26] can lead to a decrease in the efficiency of the desalination process (sharp growth of target product losses) if we consider the amino acid–mineral salt system in electrodialysis, but it can help to separate the amino acid and nonelectrolyte [30,32].

The choice of the current regime is very important and it is crucial to know the electrochemical features of amino acid solution demineralization in a wide range of current density. It is interesting to compare various types of ED and EDI cells when solving this task. The aim of this work is to analyze the electrochemical behavior of the model solution, a neutral amino acid–inorganic salt, in ED and EDI for a wide range of current densities, taking into account the influence of amino acid side radicals (aliphatic, aromatic) and the nature of mineral ions on desalination performance.

## 2. Materials and Methods

Aliphatic (α-alanine) and aromatic (phenylalanine) amino acids (AA) are considered in this research. The characteristics of them are listed in the Table 1.

**Figure 1:**
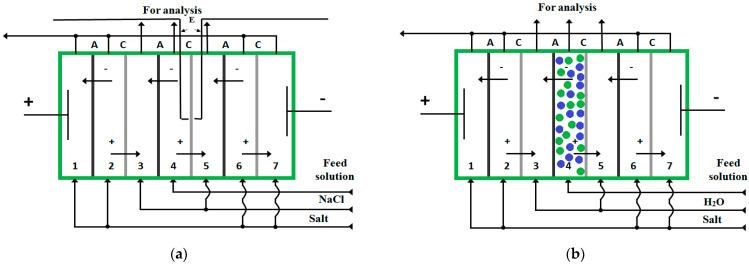
Seven-compartment electromembrane cells with alternating cation (C)—and anion (A)—exchange membranes, ‘+’—cation, ‘−’—anion, E—electrodes (AgCl), green and blue circles—anion-exchange and cation-exchange granules: (**a**) for conventional electrodialysis with smooth channel; (**b**) for EDI. (The scheme is similar for ED experiments with inert spacers in the 4th compartment but this compartment is filled with PP or styrene−divinylbenzene copolymer granules).

Electromembrane demineralization of α-alanine and phenylalanine (Sigma-Aldrich)-containing solutions is studied. The experiments have been carried out in laboratory cells (Figure 1). Both of the cells contain seven separable compartments located between two electrodes. The cathode is made of stainless steel. The anode material is platinum. The electrodialysis cell (Figure 1a) is equipped with silver chloride electrodes to analyze current–voltage characteristics of ion-exchange membranes.

In order to increase the degree of amino acid solution desalination, electrodialysis with spacers and electrodeionization procedures are used. To perform the latter, the considered dilute compartment (4) in the electrodialysis cell is filled with a mixed bed of cation- and anion-exchange resins (Figure 1b). The quantitative characteristics of performance (i.e., the degree of desalination, the target product loss) have been evaluated in conventional electrodialysis with smooth desalination channels and using various types of spacers such as monodispersed polypropylene (PP), copolymers of styrene and divinylbenzene (SDC) as well as a mixed bed of the cation-exchanger Lewatit S 1468 and the anion-exchanger Lewatit S 6328 A, Lanxess Deutschland GmbH (2:3 volume ratio, respectively).

The ED cell has alternating cation-exchange membranes (CEM) and anion-exchange membranes (AEM). Homogeneous ion-exchange membranes, Selemion CMT and Selemion AMT («Asahi Glass Co. Ltd.», Tokyo, Japan), and heterogeneous ion-exchange membranes, MA-40, MA-41 and MK-40 (LLC UCC “Shchekinoazot”, Pervomaisky, Russia), are used in this study.

The AEMs are composed of styrene-divinylbenzene co-polymers with functional quaternary ammonium groups (MA-41, AMT) and polyethylenimine with a mixture of secondary, tertiary as well as quaternary ammonium groups (MA-40). The CEMs are composed of styrene–divinylbenzene co-polymers with functional sulpho-groups. In addition, heterogeneous membranes contain polyethylene as an inert binder in their structure.

The experiments are carried out in galvanostatic mode. The flow rate of the solutions is equal to 0.1 cm·s^−1^. The test solution (amino acid (0.02 M) + mineral salt (0.01 M)) is fed into the compartment 4; 0.01 M mineral salt solution (NaCl, KCl, NaNO_3_, NH_4_Cl, or Na_2_SO_4_, ZAO Vekton) is fed into compartments 3 and 5, and mineral salt solution of a higher concentration (0.1 M) is fed into compartments 1, 2, 6 and 7. Therefore, the method of asymmetrical concentration polarization of the membranes [33] is used to control the change in the solution’s pH value in concentrate compartments and determine the value of the limiting current density for ion-exchange membranes in electrodialysis. Moreover, the nature of the cation and anion of the salt used for the feed solutions of compartments 1, 2, 6 and 7 differs from the nature of salt ions in the demineralized solution under study to reveal its specific behavior. The pH value of all the mixtures supplied into the 4th compartment is close to the isoelectric point of phenylalanine (pI = 5.91) or alanine (pI = 6.01) and ranged from 5.5 to 6.4.

The concentration of aromatic amino acids in the samples has been measured by spectrophotometry [34]; alanine, ammonium and nitrate ions have been analyzed by the photometric method. The quantitative determination of an aliphatic amino acid is based on the formation of an amino acid complex with blue-colored Cu^2+^ ions [35]. Photometric determination of ammonium ions in a sample is based on their ability to form a red−brown complex with Nessler’s reagent (an alkaline aqueous solution of potassium tetraiodomercurate (II) dihydrate K_2_[HgI_4_] · 2H_2_O) [36].

Nitrate ions were analyzed according to [37].

The content of Cl^−^-ions was detected by precipitation titration with the indicator K_2_CrO_4_ [38]. The analysis of sulfate ions was carried out by the turbidimetric method [39]. The quantitative analysis of alkali metal ions was accomplished by flame photometry [36].

## 3. ED of Solutions Containing Amino Acid and Mineral Salt Electrochemical Features

**Figure 2:**
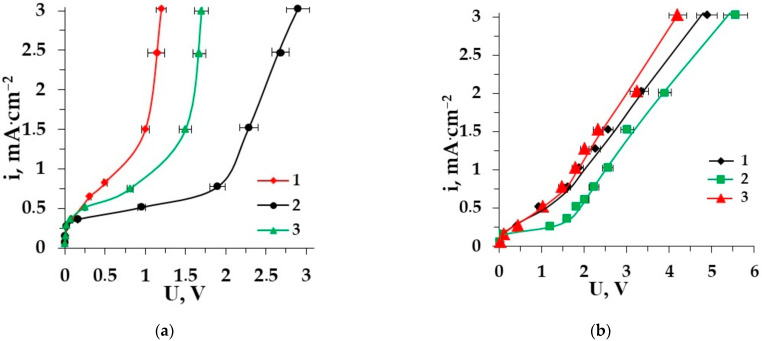
CVCs of the membrane MA-41 (**a**) and the membrane MK-40 (**b**) in the solutions: 1–Phe + NaCl, 2–NaCl, 3–Ala + NaCl.

Electrodialysis is considered a promising method for the demineralization of different multicomponent liquid media comprising AAs [40,41,42,43]. To perform the efficient ED demineralization of complex solutions, it is useful to study the electrochemical features of some model membrane systems containing AAs (zwitterlyte) along with a mineral salt (strong electrolyte). current–voltage curves (CVC) are very important characteristics for the ion-exchange membrane’s behavior. It is interesting to reveal the influence of AAs on the CVC of the membrane in a strong electrolyte solution. Figure 2 shows the difference between the CVCs of heterogeneous ion-exchange membranes for the mineral salt (NaCl) solution and for the solution mineral salt–amino acid [44,45]. Two amino acids with different side radicals (alanine and phenylalanine) are used for model solutions. Figure 2a shows the CVC for the AEM MA-41 in the solution of sodium chloride and in the solution of sodium chloride−amino acid; the CVCs for the CEM MK-40 in the same solutions are depicted in Figure 2b.

From this graph, it is clear that the CVCs for both membranes have a classical shape and consist of three regions [46]. Region I shows Ohmic behavior due to the electromigration of amino acids (if there is) and mineral salt ions at a low current density when their concentration in the boundary diffusion layer decreases with increasing voltage and current density. Then, current varies very slowly with voltage in Region II and a plateau corresponds to the limiting current density (i_lim_). The concentration of electrolyte and AA ions in the diffusion boundary layer tends to zero. Consequently, a water dissociation reaction occurs at the interphase boundary membrane solution. Region III is the overlimiting current range in which the current increases gradually. It depends on several phenomena: participation of water splitting products in the transfer of electrical charge, limiting current exaltation effect, increase in the co-ion transfer and increase in the current density along the channel due to growth of the thickness of the diffusion layer, thermal convection of the solution near a membrane, electro-osmotic flow of the solvent and electroconvection [47,48,49,50].

The comparison of three CVCs for both membranes shows that the presence of amino acids (≤0.02 M) in the salt (0.01 M) solution does not significantly affect the value of i_lim_ because the solution’s pH value is close to the isoelectric point of the amino acid. This renders most of the amino acid ions in the bipolar form so they do not take part in the current transferring through the membranes. The situation, however, changes after exceeding the limiting current density. Water splitting at the membrane–solution interface changes the pH value in the solution boundary layer as well as in the membrane phase. The bipolar ions in the membrane phase can be recharged into anions that are able to migrate through the membrane. This leads to higher values of current density at the same voltage in the system including AAs in comparison with a single salt solution [51]. The demineralization of aromatic amino acid solutions is accompanied by the increase in hydrophobicity of the membrane surface. It reduces the length of the “plateau” for the membrane CVC more significantly than the presence of aliphatic amino acids in solution.

**Figure 3:**
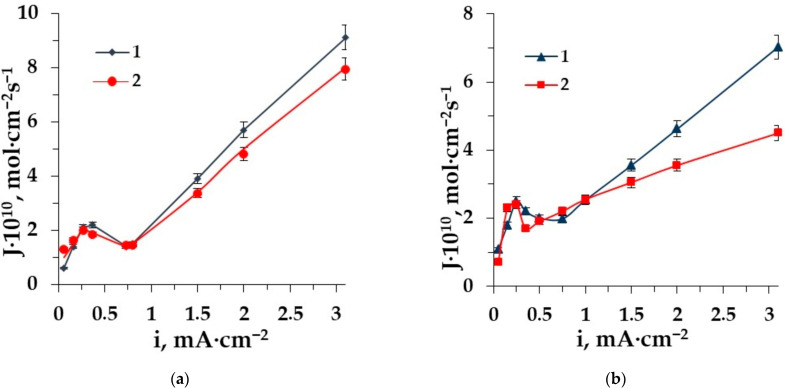
The dependence of amino acid fluxes through the membrane MA-41 (**a**) and the membrane MK-40 (**b**) on the current density in ED of solutions: 1–Ala + NaCl, 2–Phe + NaCl.

The dependence of amino acid fluxes on the current density is useful for their loss’s prognosis in ED demineralization. Figure 3a,b shows such dependences for the anion- and cation-exchange heterogeneous membranes, respectively.

The dependence of phenylalanine and alanine fluxes through the AEM and CEM on the current density have the classical shape for zwitterlytes with a maximum at the limiting current density, with a further decrease in the mass transfer due to the barrier effect [24,25] and a sharp increase when the value of the current density is 1.5–3 times more than the limiting one (facilitated transport). The maximum dependence corresponds to the limiting diffusion current density at the ion-exchange membrane CVC. Facilitated electromigration [52] of amino acids is observed in the intensive current mode and deals with the conjugative transport of amino acids with hydrogen and hydroxyl ions appearing in the course of water splitting and migrating through the following membranes in the overlimiting conditions. The facilitated transport becomes predominant because the electroconvection [53] dissipates the “barrier layers” in the desalting compartment, which restricts the transfer of amino acids through the membranes [44].

The comparison of the aliphatic and aromatic amino acid fluxes shows significant differences. The amino acid alanine flux through the membranes MA-41 and MK-40 is superior to that of phenylalanine in the studied range of current density. It is caused by the influence of the size factor of side radicals.

**Figure 4:**
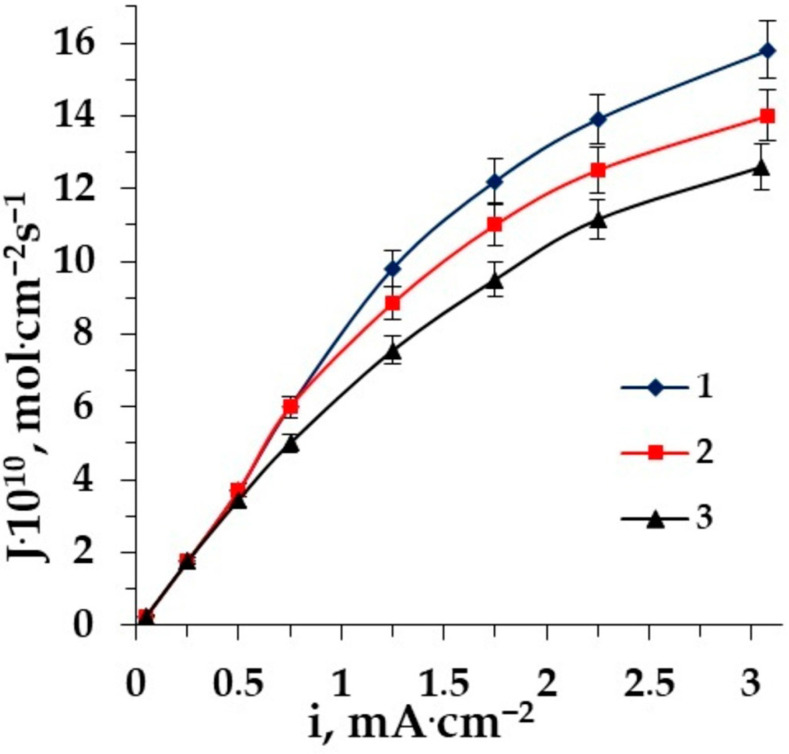
The dependence of Cl^−^-anions fluxes through the membrane MA-41 on the current density in the ED of solutions: 1–NaCl, 2–Phe + NaCl, 3–Ala + NaCl.

Besides the losses of the target product (amino acid), it is very important to predict the degree of demineralization. We have analyzed the fluxes of chlorides in the solutions: NaCl, NaCl + Ala and NaCl + Phe (Figure 4) in a wide range of current densities. The main difference is found in the overlimiting range. The additive of any AA in the solution of sodium chloride leads to a decrease in the mineral anion flux. It deals with a decrease in the mobility of chloride-ions (conjugated flux of AA) and the competitive transport of amino acids (diffusion and migration) through the membrane.

It is necessary to emphasize that type of mineral salt anion has a definite influence on AA flux through the anion-exchange membrane [54]. The influence of mineral anion type, the ion radius and hydration on the fluxes of AA through the anion-exchange membranes MA-41 in ED of solutions containing alkyl aromatic amino acids such phenylalanine and sodium chloride is studied.

**Figure 5:**
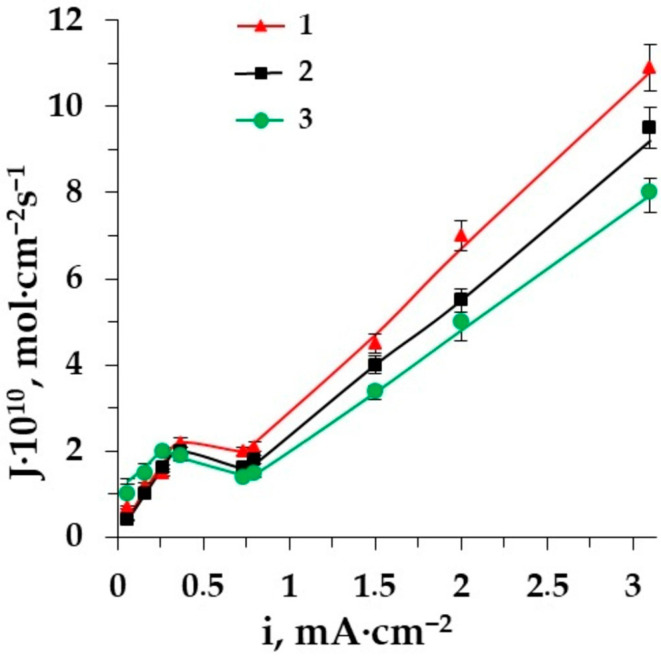
The dependence of Phe fluxes through the membrane MA-41 on the current density in ED of solutions: 1–Phe + Na_2_SO_4_, 2–Phe + NaNO_3_, 3–Phe + NaCl.

The comparison of amino acid fluxes through the membrane MA-41 has been made from the solutions NaCl–AA and NaBr–AA. The conjugative transport of AAs with bromide ion is more intensive. However, this result is interesting only in the theoretical aspect because bromides are not used in the synthesis procedure. It is more useful to compare AA fluxes from the solutions of nitrates, which are applied in the microbiological synthesis of AA as a source of nitrogen. Figure 5 compares the fluxes of phenylalanine from various mixed solutions.

The main difference in the dependence of amino acid fluxes on the current density in ED of solutions containing amino acids and mineral salts with different anions (chloride, sulfate and nitrate ions) is observed in the overlimiting conditions. The amino acid flux through the membrane MA-41 in the electrodialysis of sodium sulfate + AA solution is superior to fluxes from the other two salt solutions in the intensive current mode. The lowest amino acid flux is determined in the demineralization of the sodium chloride + AA solution. This is explained by the most pronounced competitive transport of amino acids and chloride anions.

**Figure 6:**
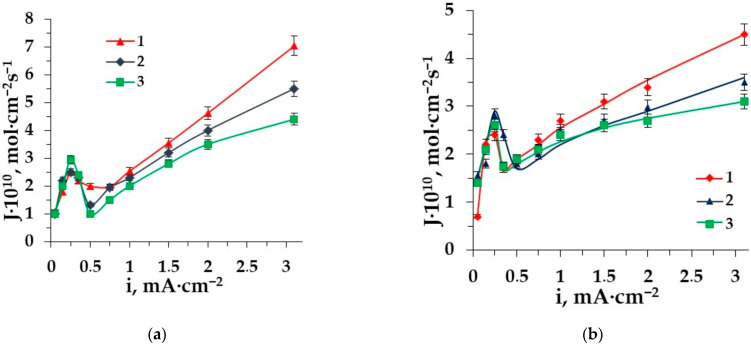
The dependence of amino acid fluxes through the membrane MK-40 on the current density in the ED of solutions: (**a**) 1–Ala + NaCl, 2–Ala + NH_4_Cl, 3–Ala + KCl; (**b**) 1–Phe + NaCl, 2–Phe + NH_4_Cl, 3–Phe + KCl.

The type of mineral salt cation also has a great impact on amino acid fluxes. We compared two solutions in our earlier work [54]. It has been found that due to the different type of hydration for sodium and potassium ions, the flux of sodium ions is higher. In this work we compare three types of cations: sodium, potassium and ammonium. The dependence of AA fluxes through the CEM MK-40 on the current density are shown in Figure 6 for various mixed solutions (a—alanine, b—phenylalanine).

The lowest amino acid flux is obtained in the ED of the solution containing potassium chloride. This can be due to the K^+^-ion’s negative hydration, resulting in a higher mobility of water molecules in the hydration shell. As it is clear from the figure, the presence of Na^+^ ions in the system promotes the transport of AA through the CEM more than the presence of K^+^ ions. The amino acid can be retained in the hydration shell of the positively hydrated Na^+^ ion and transported through the CEM after exceeding the limiting current density. There are intermediate values of the conjugative transport of amino acids with ammonium through the CEM MK-40 due to low mobility and specific properties of ammonium influencing the flux of AAs in the electrodialysis of solutions containing AAs and ammonium salt.

**Figure 7:**
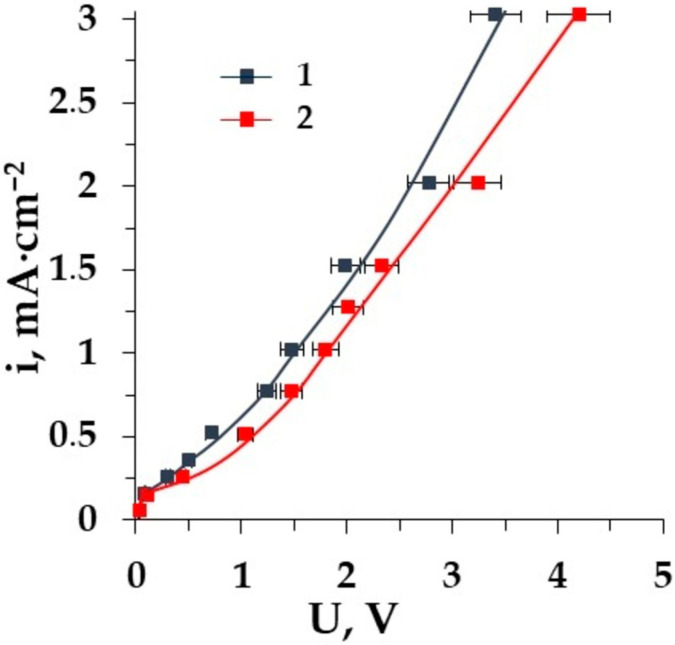
CVCs of the membranes: 1–CMT, and 2–MK-40 in solutions Ala + NaCl.

The choice of membranes for ED desalination should be done taking into consideration their electrochemical characteristics. It is shown in [44,55] that the losses of AAs that transport through the homogeneous AEM are higher. The great difference between the plateau length of the CVC for the heterogeneous and homogeneous membranes has been fixed. This value is the lowest for the homogeneous AEM. The comparison of CVCs for various cation-exchange membranes is shown in Figure 7.

The difference in the shape of the CVC for the homogeneous and heterogeneous membranes in the mineral salt solution as well as in the mixed solution of salt with the tested amino acid was detected at current densities higher than the limiting one. The value of the limiting current density is the same for both the homogeneous CMT and the heterogeneous MK-40 membranes. Membrane MK-40 and CMT have the same structure of matrix with sulpho-groups. In the case of the CMT membrane in the system, the process of water splitting is more intensive than it is with the MK-40 in the ED cell due to the surface properties of the CMT membrane, which is rather homogeneous and smooth. The difference in the structure of the membrane surface leads to the decrease in the length of the CVC “plateau” for the homogeneous membrane compared with the heterogeneous membrane [56].

**Figure 8:**
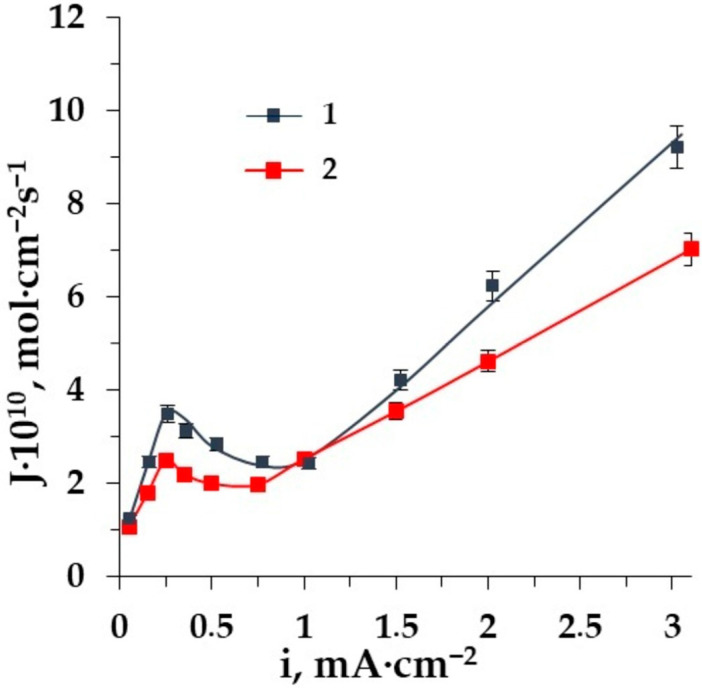
The dependence of alanine fluxes through the membranes CMT (1), MK-40 (2) on the current density in the ED of solution Ala + NaCl.

In amino acids solutions, the important task of demineralization is the choice of the membrane type, which controls the amino acid losses and demineralization degree. The comparison of the amino acid fluxes through the homogeneous and heterogeneous membranes in a wide range of current densities is shown in Figure 8.

The differences in the mass transfer of amino acids in the entire range of current densities through the homogeneous membrane AMT as compared to the heterogeneous MA-41 one are revealed. The influence of water splitting is more intensive for the homogeneous membrane AMT. This is due to the uniformity of the homogeneous membrane’s surface structure. The availability of ion-exchange fixed groups that catalyze water splitting is greater. Therefore, the expected losses of amino acids in ED demineralization are supposed to be more significant with homogeneous membranes.

## 4. Desalination of Amino Acid Solutions

The parameters that influence the process of phenylalanine and alanine solution desalination by ED and EDI are analyzed. These parameters are physical and chemical properties of the separated components (AAs and inorganic salt ions), current mode of the process, membrane structure, types of spacers in the electromembrane cell and interactions of the separated components with a membrane and an applied spacer.

The possibilities of desalination for the model solutions of the neutral AA (0.02 M), also containing a strong electrolyte (0.01 M) (sodium, ammonium or potassium chloride, sodium nitrate or sulphate), are discussed in this work. The degree of desalination and amino acid losses in its separation from the mineral salt are evaluated using pairs of heterogeneous membranes MA-41/MK-40, MA-40/MK-40 as well as the homogeneous membranes CMT/AMT.

### 4.1. The Influence of Amino Acid Side Chain Nature on the Demineralization Parameters

In the present work, the amino acid losses and the desalination degree of solutions containing amino acids with a different structure of the side chain and sodium chloride are considered in electrodialysis. The losses of the aliphatic amino acid α-alanine reach greater values (16.4–18.0%) than those of the aromatic amino acid phenylalanine (15.5–15.9%) in all studied systems with various membrane pairs in the intensive current mode. This is explained by the lower mobility of phenylalanine in a solution and in a membrane phase, due to the larger volume of its side radical compared to that of alanine. The desalination degree for solutions containing phenylalanine is greater (96.0–99.6%) than for solutions containing alanine (95.0–97.9%). Therefore, these desalination process parameters correlate with the discussed transport characteristics of the considered membranes.

### 4.2. The Influence of Mineral Ion Nature on the Demineralization Parameters

The effect of mineral ion nature on amino acid transport through the cation- and anion-exchange membranes and the following losses in the desalination process have been studied.

It has been found that in the presence of mineral salt cations with positive hydration energy, the loss of amino acids reaches the highest values at current densities above the limiting one. The negative hydration of potassium ions leads to lower amino acid losses with this ion at high current densities. 

The losses of amino acids (L) due to the fluxes through the CEM in the presence of various mineral salt cations (X, X = Na^+^, K^+^, NH_4_^+^) change in the following order: L_Na_^+^ > L_NH_4__^+^ > L_K_^+^. At the same time, the losses of amino acids through anion-exchange membranes in the presence of various mineral salt anions (Y, Y = Cl^−^, NO_3_^−^, SO_4_^2−^) can be presented as follows: L_Cl_^−^ < L_NO_3__^−^ < L_SO4_^2−^. 

In this work, the desalination degrees due to the fluxes of mineral salt ions through the cation- and anion-exchange membranes in the presence of the amino acid are also compared. 

The desalination degree of solutions for the studied anions due to the fluxes through the anion-exchange membranes increases in the order: SO_4_^2−^ < NO_3_^−^ < Cl^−^ and correlates with the mobility value order for these ions in the phase of the AEM. The order of the mobility values in solutions is reverse: NO_3_^−^ < Cl^−^ < SO_4_^2−^ [57]. 

The desalination degree of solutions for considered cations correlates with the selectivity of the cation-exchanger from which the CEMs are made with respect to the cations of mineral salts. The selectivity of the membrane increases in the order: NH_4_^+^ < Na ^+^ <K^+^ [57].

In our consideration, the value of amino acid losses in the electrodialysis of a solution containing potassium chloride is minimal for the studied systems. It is important to note that the amino acid losses in the desalination of the ammonium chloride solution is slightly higher and does not exceed 9.9% (for the alanine solution) and 7.8% (for the phenylalanine solution) at a current density of 5 mA·cm^−2^. This allows us to conclude that in order to reduce the losses of the target product (AA), it is most preferable to use this mineral salt, which is a source of nitrogen, in microbiological synthesis. At the same time, it should be noted that when using potassium chloride mineral salt, the maximal values of the desalination degree can be achieved due to the fluxes through the membranes. The desalination degree at a current density of 5 mA·cm^−2^ for a Phe + KCl solution is equal to 99.0% and 98.1% for the Ala + KCl solution when using the pair of heterogeneous membranes MA-41/MK-40.

### 4.3. The Influence of the Membrane Type used on the Desalination Process Parameters

The choice of ion-exchange membranes used for the desalination of AA solutions by electrodialysis is a task of great importance because the structure of the membrane, the type of ionogenic groups and the polymer matrix determine their characteristics. This paper attempts to determine the influence of such factors.

Amino acid losses due to mass transfer through MA-40 membranes reach the highest values in comparison with other anion-exchange membranes. This is connected with the catalytic activity of the secondary and tertiary amino groups of the membrane, which is greater than that of the quaternary ammonium groups of the AMT and MA-41 membranes. The absence of polyethylene in the structure of the AMT membrane ensures greater accessibility of the functional groups of this membrane compared to its heterogeneous analog. Therefore, the amino acid losses are observed to be greater in the electrodialysis using homogeneous AMT membranes as compared to heterogeneous MA-41 membranes. A similar feature was also revealed for the two considered CEMs.

However, the desalination degree for amino acid solutions with homogeneous membranes is the best among the considered pairs. For example, at a current density of 5 mA·cm^−2^, the desalination degree reached a maximal value equal to 99.5% for the Ala + KCl solution and 99.8% for the Phe + KCl solution.

The use of AEM MA-41 and CEM MK-40 leads to the lower values of the desalination degree. At the same time, the application of the MK-40/MA-41 membrane pairs with the electrodialysis of the AA + mineral salt solution, making it possible to reduce amino acid losses, which reach 8.5% (for alanine + KCl solution) and 7.5% (for a phenylalanine + KCl solution) at a current density of 5 mA·cm^−2^.

### 4.4. The Influence of the Spacer Type used on the Desalination Parameters

To intensify the mass transfer of ions through the ion-exchange membranes from the solution in the desalination compartment, various types of spacers (non-conducting spacers, ion-conducting granules [58,59], ion-exchange membrane nets [60]) are applied. The literature presents a restricted number of works in which the method of EDI is used for the desalination of solutions containing amino acids: neutral [61], acidic [62] and basic [63].

The desalination of the phenylalanine solution by conventional electrodialysis, electrodialysis with inert granulated polypropylene spacers, styrene−divinylbenzene copolymers (SDS) and ion-conducting spacers, such as a mixed bed of strong acidic cation-exchanger S 1468 and strong basic anion-exchanger S 6328 A in a volume ratio of 2:3, respectively, is compared in this study based on the results of our earlier work [64] and new experimental dependences. 

The effects observed in the transport of organic ampholytes influences the process of their separation with strong electrolytes. The desalination of solutions containing amino acids at current densities corresponding to the area of the barrier effect action is accompanied by low amino acid losses. In the area of action of the barrier effect, amino acid losses are significantly reduced; in particular, in the electrodialysis of a Phe + NaCl solution, the losses decrease by 2.4 times and do not exceed 2.0% at a current density of 0.36 mA cm^−2^.

**Figure 9:**
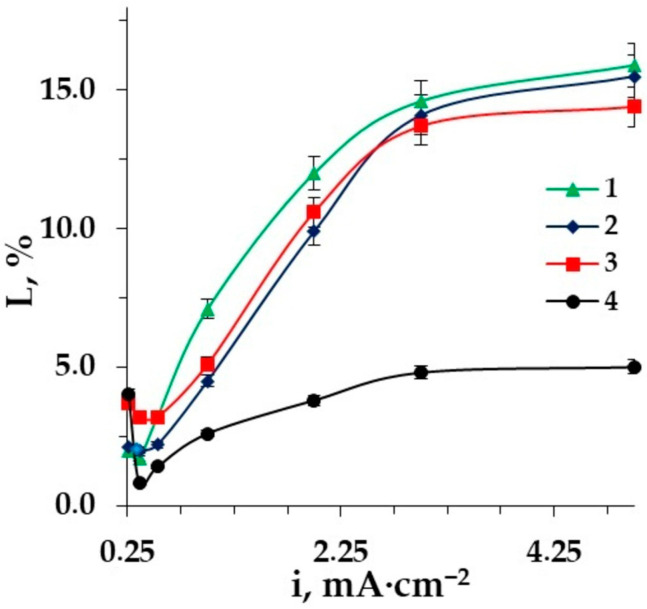
The dependence of amino acid losses on the current density in the desalination of Phe (0.02 M) + NaCl (0.01 M) solution: 1–ED with styrene−divinylbenzene granulated copolymer; 2–ED; 3–EDI; 4–ED with granulated polypropylene as a spacer.

**Figure 10:**
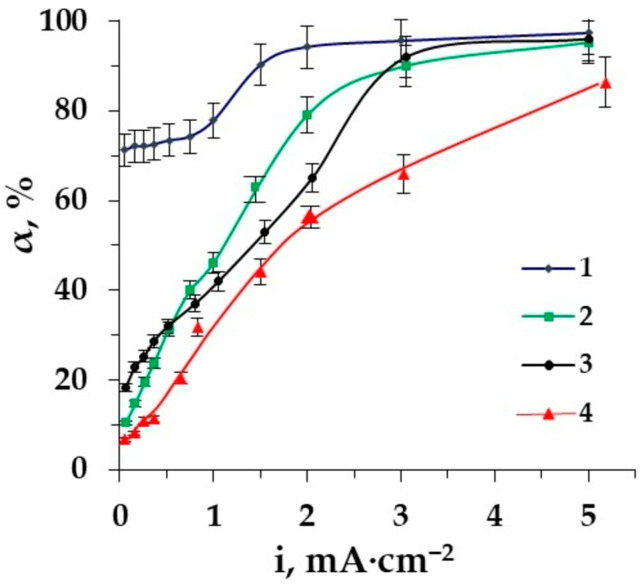
The dependence of the desalination degree of Phe (0.02 M) + NaCl (0.01 M) solution on the current density in the electromembrane system: 1–EDI, 2–ED with styrene−divinylbenzene granulated copolymer, 3–ED with granulated polypropylene as a spacer, 4–ED.

In the intensive current regime, amino acid fluxes increase significantly due to the effect of facilitated electromigration—the conjugated transport of amino acids and OH^−^ and H_3_O^+^- ions. The losses of the target desalination product grow accordingly. However, the degree of desalination reaches the highest values in a more intensive current mode. The dependences of amino acid loss and desalination degree for various types of spacers on the current density are shown in Figure 9 and Figure 10.

**Figure 11:**
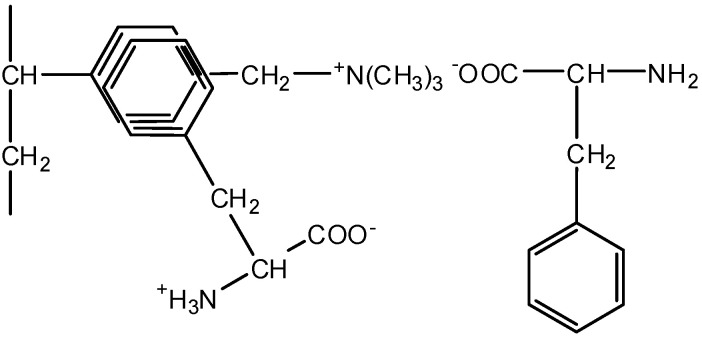
Scheme of the interactions of phenylalanine benzene ring with the aromatic fragment of styrene−divinylbenzene copolymer of anion-exchange membrane.

The use of inert spacers makes it possible to increase the degree of desalination in comparison with conventional ED with smooth desalination channels. Moreover, in the desalination of aromatic amino acid solutions, the use of polypropylene is more rational than the use of the styrene−divinylbenzene copolymer, because in this case, amino acid losses are reduced by 3.2 times to a minimum which is equal to 5.0% at the current density 5 mA·cm^−2^. The structural similarity of aromatic fragments of phenylalanine and styrene−divinylbenzene copolymers leads to the larger losses of the target product in desalination due to the additional interactions between benzene rings of Aas and the matrix (stacking effect), that is, π-π interactions [65]. A scheme of the interactions of the phenylalanine benzene ring with an aromatic fragment of the styrene−divinylbenzene co-polymer of AEMs with functional quaternary ammonium groups (MA-41, AMT) is shown in Figure 11.

Rather high values of the desalination degree in the demineralization of amino acid–mineral salt solutions by the method of electrodeionization are already reached at low current densities. The degree of desalination equal to 72.1% was already achieved at a current density of 0.26 mA·cm^−2^. This makes it possible to reduce energy consumption in the purification of the amino acid solution from mineral salts. At the same time, the maximum values (97.3%) of the desalination degree are observed in the intensive current mode.

The use of ion-conducting spacers seems to be promising, since the highest values of the desalination degree are achieved both in the underlimiting and in the intensive current mode of EDI; however, it leads to the maximum losses of amino acid. The application of a spacer such as granulated polypropylene is reasonable if it is necessary to reduce the loss of the target product.

The current mode for separation of zwitterlytes and mineral salts can be chosen depending on the requirements for the target product. The action of the barrier effect can be used to reduce amino acid losses but the desalination degree in this range is rather low. If it is necessary to reach deep desalination, the intensive current regime is required but its drawback for the process is a growth of AA losses.

## 5. Conclusions

The transport characteristics and current–voltage curves of different ion-exchange membranes in the ED of solutions containing neutral amino acids and mineral salts are analyzed. The influence of the amino acid side chain structure and the nature of mineral salt ions on the parameters of the demineralization process are evaluated. The choice of the ion-exchange membranes and spacer type is also a key factor. Taking into account the structure of the side chain of the amino acid, its mobility, as well as the nature of the mineral salt ions, their hydration energy and other properties, it is made possible to predict the efficiency of the deionization of the mixed solution.

In particular, the demineralization of aromatic amino acid (phenylalanine) solutions is accompanied by an increase in the hydrophobicity of the membrane surface. This reduces the “plateau” length of the membrane CVC more significantly than the presence of aliphatic amino acids (alanine) in solution. The alanine flux through the ion-exchange membranes is superior to that of phenylalanine in studied range of current density. The losses of amino acids caused by their fluxes through AEMs in the presence of various mineral salt anions can be presented as follows: L_Cl_^−^ < L_NO3_^−^ < L_SO4_^2−^. Moreover, the desalination degree of solutions for the considered salts with various cations correlates with the selectivity of the cation-exchanger from which the CEMs are made with respect to the cations. The losses of the amino acids caused by their fluxes through the CEM in the presence of various mineral salt cations decrease in the following order: L_Na_^+^ > L_NH4_^+^ > L_K_^+^.

The desalination degree for amino acid solutions in the ED with homogeneous membranes is the best among the considered membrane pairs. The desalination degree is equal to 99.5% for the Ala + KCl solution and 99.8% for the Phe + KCl solution at the current density of 5 mA·cm^−2^. The application of the MK-40/MA-41 membrane pairs in electrodialysis allows for a reduction of amino acid losses, which reaches 8.5% for the Ala + KCl solution and 7.5% for the Phe + KCl solution at a corresponding current density. 

It is important to note that amino acid losses in the desalination of the ammonium chloride-containing solution is slightly higher and does not exceed 9.9% for the Ala-containing solution and 7.8% for the Phe-containing solution in considered conditions.

The choice of membranes (functional groups and the structure of polymeric matrix) is important to control and regulate the mass transfer of components. To achieve the higher desalination degree of amino acid solutions, the most promising method is EDI. At the same time, the use of polypropylene granulated spacers in the electrodialysis of the amino acid–mineral salt solution makes it possible to reduce losses of the target product. 

## Figures and Tables

**Table 1:** Some properties of studied amino acids.

Amino Acid	Structural Formula	pI	pK		Molecular Weight	Solubility, g/100 mL H_2_O, 25 °C	Side Radical Volume, nm^3^
			pK_1_	pK_2_			
Alanine α-Ala	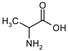	6.01	2.34	9.69	89.09	16.65	0.0322
Phenylalanine Phe	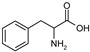	5.91	2.58	9.24	165.19	2.96	0.1366

## Data Availability

Not applicable.
